# Activation of Th2 cells downregulates CRTh2 through an NFAT1 mediated mechanism

**DOI:** 10.1371/journal.pone.0199156

**Published:** 2018-07-03

**Authors:** Emily MacLean Scott, Lauren A. Solomon, Courtney Davidson, Jessica Storie, Nami Shrestha Palikhe, Lisa Cameron

**Affiliations:** 1 Pulmonary Research Group, Department of Medicine, University of Alberta, Edmonton, Alberta, CANADA; 2 Department of Pathology and Laboratory Medicine, Western University, London, Ontario, CANADA; Instituto Nacional de Cancer, BRAZIL

## Abstract

CRTh2 (encoded by *PTGDR2)* is a G-protein coupled receptor expressed by Th2 cells as well as eosinophils, basophils and innate lymphoid cells (ILC)2s. Activation of CRTh2, by its ligand prostaglandin (PG)D_2_, mediates production of type 2 cytokines (IL-4, IL-5 and IL-13), chemotaxis and inhibition of apoptosis. As such, the PGD_2_-CRTh2 pathway is considered important to the development and maintenance of allergic inflammation. Expression of CRTh2 is mediated by the transcription factor GATA3 during Th2 cell differentiation and within ILC2s. Other than this, relatively little is known regarding the cellular and molecular mechanisms regulating expression of CRTh2. Here, we show using primary human Th2 cells that activation (24hrs) through TCR crosslinking (αCD3/αCD28) reduced expression of both mRNA and surface levels of CRTh2 assessed by flow cytometry and qRT-PCR. This effect took more than 4 hours and expression was recovered following removal of activation. EMSA analysis revealed that GATA3 and NFAT1 can bind independently to overlapping sites within a *CRTh2* promoter probe. NFAT1 over-expression resulted in loss of GATA3-mediated *CRTh2* promoter activity, while inhibition of NFAT using a peptide inhibitor (VIVIT) coincided with recovery of CRTh2 expression. Collectively these data indicate that expression of CRTh2 is regulated through the competitive action of GATA3 and NFAT1. Though prolonged activation led to NFAT1-mediated downregulation, CRTh2 was re-expressed when stimulus was removed suggesting this is a dynamic mechanism and may play a role in PGD_2_-CRTh2 mediated allergic inflammation.

## Introduction

CRTh2 (chemoattractant receptor homologous molecule expressed on Th2 cells) is a seven transmembrane spanning receptor for prostaglandin D_2_ (PGD_2_) [1], a lipid mediator released from allergen/IgE activated mast cells [2] and macrophages following microbial activation [3]. Activation of CRTh2 (encoded by *PTGDR2)* mediates chemotaxis [1], production of pro-allergic cytokines [1, 4, 5] and inhibition of apoptosis [6]. CRTh2 expression by human CD4^+^ T helper lymphocytes is considered the most reliable marker of Th2 cells [7–11], but CRTh2 is also expressed by eosinophils, basophils [11, 12] and a subset of innate lymphoid cells (ILC2) [13]. Together Th2 cells and ILC2s orchestrate development of allergic inflammation through production of IL-4, IL-5 and IL-13 [14, 15] which induces production of IgE, inflammatory cell infiltration to sites of exposure and tissue remodeling [16].

The importance of the PGD_2_-CRTh2 pathway to the development and maintenance of allergic inflammation has been substantiated with animal and human studies. Over-expression of PGD_2_ synthase (PGDS) [17] or use of CRTh2 agonists enhanced eosinophilia and type 2 cytokine release in the airways of allergen-challenged animals [18]. Mice made genetically deficient of CRTh2 showed reduced skin [19, 20] and nasal mucosal infiltration of eosinophils and production of type 2 cytokines [21] as well as a sustained reduction in eosinophil accumulation in the airways in a chronic model of asthma [22]. Similarly, CRTh2 antagonists have been shown to reduce eosinophil accumulation, type 2 cytokine and IgE production in the airways [23] and skin [24] of animal models of allergic disease. In humans, expression of CRTh2 is higher in the skin of patients with atopic dermatitis [14] and the airways of patients with asthma [25, 26]. We showed that the proportion of circulating CD4^+^CRTh2^+^ T cells (*i*.*e*., Th2 cells) are elevated in subjects with allergic airways disease compared to non-allergic controls [27] and are associated with asthma severity as they are higher in those classified with severe compared mild/moderate disease [28]. In light of this body of work, a number of CRTh2 antagonists are in clinical trials and showing this approach to have therapeutic potential [29, 30].

Persistence of allergic disease is mediated by an adaptive immune response whereby T and B cells develop memory to the allergen. Th2 cells isolated from blood have been shown to be central memory T cells (T_CM_), expressing CD62L and CCR7 [8, 9, 31]. The role of T_CM_ cells is to circulate within the body and rapidly move to the periphery upon allergen exposure, where they interact with dendritic cells, undergo clonal expansion and generate a population of T effector cells [32]. Emigration of T_CM_ cells out of tissues and back to secondary lymph nodes occurs when the allergen is cleared [33, 34]. This process maintains a pool of memory T cells for rapid response to subsequent exposures, advantageous when fighting a pathogen or maintaining immunity developed from vaccination. In the case of allergic responses, however, T_CM_ cells are deleterious as they are responsible for the persistent memory to allergen. CRTh2 may be involved in this persistence, since a recent study showed that it mediates exit of T_CM_ cells from inflamed tissues via the lymphatic vessels [35].

Th2 cell differentiation commences when naïve CD4^+^ T cells are activated by antigen-induced T cell receptor (TCR) crosslinking. This results in activation of nuclear factor of activated T cells (NFAT) and activator protein (AP)-1 and low level IL-4 expression [36]. During the reinforcement stage, TCR and IL-4R signaling activate STAT6 and NFκβ, respectively, to upregulate GATA3 [37, 38], further increase IL-4 production [39] and remodel the chromatin at Th2 regulatory regions [40]. In humans, CRTh2-expressing Th2 cells exhibit high level GATA3 expression [41] and represent terminally differentiated Th2 cells [31].

In terms of CRTh2 regulation, GATA3 over-expression has been shown to induce CRTh2 by naive human T cells [42] and also to drive CRTh2 promoter activity [43]. However, little else is known regarding the molecular processes governing its expression. Here, we report that both surface and transcript levels of CRTh2 were reduced following TCR activation of Th2 cells. GATA3 and NFAT1 both bound to the *CRTh2* promoter, but NFAT1 binding predominated following activation, when surface CRTh2 expression was lowest. Over-expression of NFAT1 interfered with GATA3 induction of *CRTh2* promoter activity, while inhibition of NFAT nuclear translocation resulted in recovery of CRTh2 expression. Collectively, these data show that CRTh2 is regulated by TCR activation and suggest a mechanism by which NFAT1 inhibits GATA3-mediated expression. Re-expression of CRTh2 following removal from activation indicates this is a dynamic process that could participate in the maintenance of memory Th2 cells.

## Materials and methods

### Cell lines and *in vitro* differentiated human Th2 cells

Jurkat cells (clone E6-1) were purchased from American Type Culture Collection (VA, USA) and cultured in RPMI 1640 media (Sigma Aldrich, ON, Canada) supplemented with Fetal Bovine Serum (10%; Hyclone Scientific, Fisher Scientific, Ontario, Canada) and penicillin, streptomycin and glutamine (1X; Gibco, ON, Canada). Peripheral blood mononuclear cells (PBMCs) were isolated from healthy donors by density centrifugation over Ficoll Histopaque PLUS (GE Healthcare, Sweden) and CD4^+^ T cells were isolated by negative selection (CD4^+^ T cell Isolation Kit II, Miltenyi Biotech, CA, USA). CD4^+^ T cell purity was > 96%. Cells were primed on plate bound antibody (anti-) to CD3 (Clone UCHT1, 1 μg/mL) and anti-CD28 (Clone 37407, 1 μg/mL) in Th2 differentiating conditions; recombinant human (rh) IL-2 (5 ng/mL), rhIL-4 (10 or 20 ng/mL) and blocking antibodies against IFNγ (1μg/mL) and IL-12 (1μg/mL) for 3 days in X-VIVO 15 medium (Lonza, USA) supplemented with 10% fetal bovine serum (Hyclone) and 1% penicillin/streptomycin/glutamine (Gibco, Canada). On day 4, cells were re-plated and rested with cytokines and blocking antibodies but without activating CD3 and CD28. After 7 days of differentiation, CRTh2^+^CD4^+^ T cells were isolated by positive selection (CRTh2^+^ cell selection kit, Miltenyi Biotech, CA, USA). CRTh2^+^CD4^+^ T cells were maintained on cycles of activation (3 days αCD3/CD28 + IL-2) and rest (4 days IL-2). Experiments were performed between days 10 and 60, when CRTh2 expression was ≥ 50%. To examine recovery of CRTh2 expression following activation, *in vitro* differentiated CRTh2^+^CD4^+^ T cells were activated on plate bound anti-CD3/CD28 (24 hours) then washed, re-plated in IL-2 and examined (6–96 hours). Recombinant human cytokines and monoclonal antibodies were all from R&D Biosystems Inc. (MN, USA) except antibody against IL-12, which was from eBiosciences Inc. (CA, USA). For some experiments, activation with phorbol 12-myristate 13-acetate (PMA) (20 ng/mL, Sigma Aldrich, ON, Canada) and ionomycin (Iono; 1μM, Sigma Aldrich, ON, Canada) was used. Where indicated, NFAT activation was inhibited with 11-R-VIVIT (cat# 480401, Calbiochem, Germany). The control peptide 11R-VEET was custom synthesized (EZbiolab, IN, USA) [44]. This study was approved by the University of Alberta Human Ethics Review Board (approval number 00000942).

### Flow cytometry

All antibodies were purchased from BD Pharmingen (Mississauga, ON, Canada), unless otherwise indicated. Isolated CD4^+^ T cells were stained with antibody against CD4 (Clone RPA-T4, AbD Serotech) or isotype control and CD45RA to determine purity of the naïve CD4^+^ T cell population (>96%). *In vitro* differentiated Th2 cells were stained for CRTh2, CD62L and CD45RO. Cells were blocked with normal rat IgG **(**Invitrogen, CA, USA**)** at room temperature (30 min) and stained with fluorochrome-conjugated primary antibody (CD62L, CD45RO) or isotype control. For CRTh2, a biotin-conjugated anti-CRTh2 antibody (Clone BM16, Miltenyi biotech, CA, USA) or biotinylated rat IgG2a isotype (ABD Serotech, NC, USA) was used. Samples were incubated at 4°C (30 min) followed by Streptavidin-APC (eBioscience, CA, USA), at 4°C (30 min) and fixed (2% paraformaldehyde + 0.54% sucrose). Intracellular cytokine staining for IL-4, IL-13 and IFNγ was performed following 4 hours of stimulation with PMA (20ng/mL, Sigma Aldrich, Canada) and Iono (1 μM, Sigma Aldrich, Canada) in the presence of Brefeldin A (10 μg/mL, Sigma Aldrich, Canada) for intracellular sequestration of cytokines. Cells were fixed on ice (10 min) with paraformaldehyde (4%; Sigma Aldrich, Canada) and permeabilized on ice (10 min) with saponin (0.4%, Sigma Aldrich, Canada). IL-13-PE (Clone JES10-5A2, isotype rat IgG1κ PE), IFNγ-Alexa 647 (Clone B27, isotype mouse IgG1κ Alexa 647) and IL-4-Alexa 488 (Clone 8D4-8, isotype mouse IgG1κ Alexa 488) antibodies (BD Pharmingen, ON, Canada) were added and samples were incubated at 4°C (30 min) and after washing cells. Fluorescence was assessed immediately using a FACS Calibur (Becton Dickson, ON, Canada) and data analyzed using FlowJo (Tree Star Inc, Ashland, OR, USA). Percentage of positive cells were determined by setting gates on isotype control.

### Sequence analysis

*PTGDR2* sequence was obtained from Ensembl (http://www.ensembl.org) and *in silico* analysis of transcription factor binding sites was performed using MatInspector (www.genomatix.de). Matrix search parameters set at a core similarity of 0.75 and an optimized matrix similarity.

### Nuclear extracts and recombinant proteins

Nuclear extracts were made from Jurkat T cells in log phase growth (12 x 10^6^ cells) and *in vitro* differentiated CRTh2^+^ CD4^+^ T cells (12 x 10^6^ cells). Cells were either stimulated with PMA (20ng/mL) and ionomycin (1 μM; 3h) or left unstimulated in media alone (3h). All subsequent steps were performed on ice. Cells were harvested and resuspended in lysis solution (Buffer A: 10 mM HEPES, 3 mM MgCl_2_, 40 mM KCl, 1 mM DTT, 5% glycerol, 0.20% NP-40, 1 mM PMSF, 5 mM β-glycerophosphate, 1 mM benzamidine, 1 mM sodium orthovanadate, 1 mM sodium fluoride, 10 μg/mL antipain, 10 μg/mL aprotinin, 10 μg/mL leupeptin, 10 μg/mL pepstatin: 120 μL). Lysis was assessed with trypan blue. Cell nuclei were pelleted (4°C, 13,200 RPM). Nuclear pellets were resuspended in an appropriate volume of Buffer C (420 mM NaCl, 10 mM HEPES, 25% glycerol, 1.5 mM MgCl_2_, 0.2 mM EDTA, 1.0 mM DTT, 1 mM PMSF, 5 mM β-glycerophosphate, 1 mM benzamidine, 1 mM sodium orthovanadate, 1 mM sodium fluoride, 10 μg/mL antipain, 10 μg/mL aprotinin, 10 μg/mL leupeptin, 10 μg/mL pepstatin). PMSF and DTT were added to Buffer A and C immediately before use. Nuclear extracts were flash frozen with liquid nitrogen and stored at -80°C until use. Complementary (c) DNA for GATA3 and NFAT1 were sub-cloned into pcDNA3.1^+^ (Invitrogen, ON, Canada). Recombinant human GATA3 and NFAT1 proteins were synthesized from these vectors by *in vitro* transcription and translation (TnT), with the TnT coupled rabbit reticulocyte lysate system and T7 polymerase according to manufacturers’ instructions (Promega, WI, USA). Plasmid (1μg) was incubated with rabbit reticulocyte lysate (25 μl), reaction buffer (2 μl), T7 polymerase (1 μl), leucine (0.5μl) and methionine (0.5μl) in a final reaction volume of 50μl in water. Reactions were incubated in a 30°C water bath (90 min) and lysates were stored at -80°C.

### Electromobility shift assay (EMSA)

Gel shift assays were performed with an oligonucleotide probe (5’ CGGCATTGATGGAAATTGATGATATTTGAAC) representing position -125/-94 of the *CRTh2* promoter region (relative to the transcription start site). Annealed oligonucleotides (100 ng) were 5’ end labeled with [γ-^32^P]–ATP using T4 polynucleotide kinase (New England Biolabs, ON, Canada) and excess radioactivity was removed using a Mini Quick Spin DNA column (Roche, Sweden). Labeling efficiency was measured by scintillation (counts per minute per μl). Probes were stored at -20°C and used within one week. The EMSA binding reaction included nuclear extract (5 μg) or recombinant protein (1 μL of TnT reaction), 50 mM NaCl, 10% glycerol, poly dI:dC (50 ng/μL, Sigma Aldrich, ON, Canada), BSA (0.2 μg/μL) and 1X protease inhibitor (Roche, IN, USA). Nuclear extracts or recombinant proteins were incubated on ice (30 min) in the described binding conditions; either alone, with super-shifting antibodies (4 μg) or with 100 molar excess (100X) of unlabeled *CRTh2* proximal promoter oligonucleotide, consensus probes for transcription factors or mutants of the *CRTh2* proximal promoter oligonucleotide (Table 1). To mutate transcription factor binding sites, oligonucleotides were generated with transversions (purine to pyrimidine or vice versa) in the core binding sites. Most super-shifting antibodies were purchased from Santa Cruz Biotechnology (CA, USA); GATA3 (HG3-31 and HG3-35), NFAT1 (4G6-G5), isotype matched control antibody (ATF2; Clone F2BR-1). The super-shifting antibody for NFAT2 (MA3-024) was purchased from Affinity Bioreagents (CO, USA). Probes were then incubated on ice (30 min) with the nuclear extract binding reactions as described above. Binding reactions were loaded onto a 5% non-denaturing polyacrylamide gel and run at 19 mA for 5–6 hours with 0.5X Tris-buffered EDTA at 4°C. Gels were dried at 80°C (1h) (Hoefer Slab Gel Dryer, GD2000) and exposed to film for up to 48 hours at -80°C.

**Table 1:** EMSA oligonucleotide sequences.

Name	Site(s) Mutated	Sequence
NFAT consensus		5'CGGAGGAAAAACTGTTTCATACAGAAGGCGTG
GATA3 consensus		5'CCTCTATCTGATTGTTAGCC
CRTh2 proximal		5'CGGCATTGATGGAAATTGATGATATTTGAAC
NFAT1.1	N1	5'CGGCATTGAT**cctt**ATTGATGATATTTGAAC1
GATA3-m1	G1	5'CGGCATT**c**ATGGAAATTGATGATATTTGAAC1
GATA3-m2	G2	5'CGGCATTGATGGAAATT**c**ATGATATTTGAAC1
GATA3-m3	G3	5'CGGCATTGATGGAAATTGATGA**a**ATTTGAAC1
GATA3-m1+ m3	G1/G3	5'CGGCATT**c**ATGGAAATTGATGA**a**ATTTGAAC1

^1^Lower case letters indicate transversion mutations

### Transient transfection

A CRTh2 luciferase reporter construct (*CRTh2*pro/Luc) was generated by cloning the region 450 base pairs upstream of the transcription start site (TSS) of CRTh2 into pGL3 basic construct as previously described [43] (Promega, WI, USA). Site-directed mutagenesis was conducted on the CRTh2pro/Luc vector to delete position -125/-94 of the CRTh2 promoter (Forward 5’ ATCTGTGTGGCAACTCACTCTCCAG 3’, Reverse 5’ GCAGCCCCTGCTCCACCC 3’). PCR was conducted using a Q5 Site-Directed Mutagenesis Kit (New England Biolabs, USA) according to manufacturer’s instructions. Resultant colonies were screened be restriction digest with KPNI/NHEI and validated by Sanger sequencing. Jurkat T cells (5 x 10^6^) in log phase growth were transiently transfected with *CRTh2*pro/Luc (10 μg) and pRL-TK (5 ng) using electroporation. *CRTh2*pro/Luc ± expression plasmids or control DNA were added and rested on ice (10 min). Cells were electroporated with 1 pulse (240 mV) using a square wave electroporator (ECM 830, Harvard Apparatus, MA, USA). Cells were rested on ice (15 min) before incubating in media alone or with stimulus (16h, PMA; 20 ng/mL and ionomycin; 1 μM). For over-expression experiments, expression vectors were added at molar equivalents relative to GATA3; GATA3 (5 μg), pcDNA3.1^+^ (3.95 μg) and/or NFAT1 (6.1 μg or serial dilutions: 1.53, 0.76 μg). Furthermore, in order to add equal amounts of plasmid DNA during the over-expression assay, pcDNA3.1^+^ was added to co-transfection experiments to control for the total amount of DNA. Cells were harvested and lysed in 250 μl respectively, of passive lysis buffer (1X PLB, Promega, WI, USA) and frozen and thawed to increase lysis. 10 μl of lysate was added to 50 μl of Firefly luciferase substrate (luciferin) followed by addition of 50 μl of Renilla luciferase substrate (colenterazine). Total protein in each lysate was quantified using a BCA assay (Thermo scientific, IL, USA) and 20 μl of lysate. Transfection results were normalized for transfection efficiency (as determined by *Renilla* luciferase activity) and protein concentrations and expressed as relative luciferase activity (RLA). (Firefly luciferase count * (mean baseline Renilla / Renilla count) * (mean protein / protein count)).

### Quantitative reverse transcription polymerase chain reaction (qRT-PCR)

Total RNA was extracted from frozen cell pellets (2 - 10x10^6^ cells) using the RNeasy Mini kit and QIAshredders (Qiagen, Canada) and cDNA synthesized with 1 μg RNA using dNTPs, oligodT primers and SuperScriptII (Invitrogen, USA). CRTh2 mRNA was quantified using TaqMan gene expression assays for CRTh2 (Hs00173717_m1) was purchased from Applied Biosystems (Applied Biosystems, CA, USA) and used as per manufacturer’s instructions. All TaqMan probe based real time qRT-PCRs were standardized to GAPDH using primers: Forward 5’ CTGAGAACGGGAAGCTTGTCA 3’ and Reverse 5’ GCAAATGAGCCCCAGGGTT 3’. A specific probe, recognizing the GAPDH PCR product, was designed and custom ordered from ABI, 5’ 6FAMAAA TCCCATCACCATCTTCCAGGAGCGA TAMRA 3’. qRT-PCRs were performed using TaqMan gene expression master mix (Applied Biosystems, CA, USA), 1 μl cDNA (final reaction volume: 20μl) and an Eppendorf RealPlex 4 machine with the following program; 2 min 50°C, 10 min 95°C and 40 cycles of 15 sec 95°C and 1 min 60°C. All samples were run in triplicate. CRTh2 qRT-PCRs were run with a CRTh2 and GAPDH standard curve to obtain copy number. To make standards, PCR products were cloned into the pCR2.1TOPO vector (Invitrogen, CA, USA) and the vectors were linearized. The DNA concentration following purification of the linearized vector was determined by spectrophotometry and dilutions of 10^1^ to 10^9^ copies/μl were made and stored at -80°C. IL-13 qRT-PCR was performed using the TaqMan kit (Hs00174379_m1) and GAPDH primers (as above) and quantification using the ΔΔCT method.

### Chromatin Immunoprecipitation (ChIP)

Primary Th2 cells were collected after the resting phase and stimulated with PMA (20 ng/mL, Sigma Aldrich, ON, Canada) and Iono (1μM, Sigma Aldrich, ON, Canada) or left in media alone. Cells were fixed in 1% formaldehyde for (10 min) and the reaction stopped with a final concentration with 0.125M glycine (5 min). Cells were washed three times in ice-cold PBS and snap-frozen in pellets (20 x 10^6^ cells). Pellets were lysed in ChIP lysis buffer (10 mM PIPES, 10 mM KCl, 0.5% NP40, 0.1mM EDTA & EGTA) with 1mM DTT and 0.5 mM PMSF on ice (15 min). Nuclei were pelleted and lysed in ChIP nuclear lysis buffer (50 mM Tris-Cl, 10 mM EDTA, 1% SDS) with 0.02μg/mL Leupeptin & Aprotinin and 0.5 mM PMSF on ice (10 min). The lysate (0.5 ml) was sonicated on BioRuptor (Diagenode) (35 cycles, 30 seconds per pulse, 30 seconds cooling between pulses) and collected as input sample to test DNA shearing. Protein G Dynabeads (Invitrogen, CA, USA) were washed in PBS-T and pre-incubated with 10μg target antibody for 20 minutes at room temperature with rotation. Antibodies used were anti-NFAT1 (Ab2722, Abcam, Cambridge, MA, USA), anti-GATA3 (sc-269, Santa Cruz Biotechnology, CA, USA) and mouse IgG (M5284, Millipore, USA). Beads were washed in 200ul PBS-T and incubated overnight with 100μl sonicated chromatin in 900μl immunoprecipitation buffer (16.7mM Tris-HCL, 167mM NaCl, 1.2mM EDTA, 1% Triton-X100, 0.01% SDS) and incubated at 4°C overnight with rotation. Beads were washed with 1ml each Low Salt Wash Buffer (2mM Tris pH8, 1% Triton-X, 1% SDS, 167mM NaCl) (1X), High Salt Wash Buffer Buffer (2mM Tris pH8, 1% Triton-X, 0.1% SDS, 2mM EDTA, 0.5M NaCl) (1X), LiCl Wash Buffer (10mM Tris pH 8, 0.25M LiCl, 1% NP-40, 1% Sodium Deoxycholate, 1mM EDTA) (1X) and TE Buffer (10mM Tris, pH 8, 1mM EDTA) (2X). Chromatin was eluted with 1% SDS and 0.1M NaHCO_3_ (3X; 15 min) and crosslinks were reversed with 0.2 M NaCl (overnight, 65°C). DNA was purified using a Qiagen PCR purification kit and run on a gel to ensure shearing was between 200–1000bp. Quantitative PCR was done using SsoFast EvaGReen Supermix (Biorad, CA, USA). Primers used were for CRTh2 (F-GCATTGATGGAAATTGATGA; R-AACTTTGCCTTCTTC TGTGG) and IL-13 (F-GGAGCTCAGAGTTGGGTCAG; R-GCGTCTTGTGGCAGCTTTT). Negative control for ChIP was IP using mouse IgG. Data were analyzed using ΔΔCt method. Delta Ct for each sample was calculated as CTinput-CTpulldown and the Δ-Δ Ct was the Δ Ct of IgG subtracted from Δ Ct of target antibody.

### Statistical analysis

For over-expression experiments and flow cytometry, statistical significance was determined by ANOVA with post hoc analysis using Holm-Sidak method or Student’s t-test. Differences were considered significant with p<0.05.

## Results

### Generating a line of CRTh2 expressing cells

**Fig 1:**
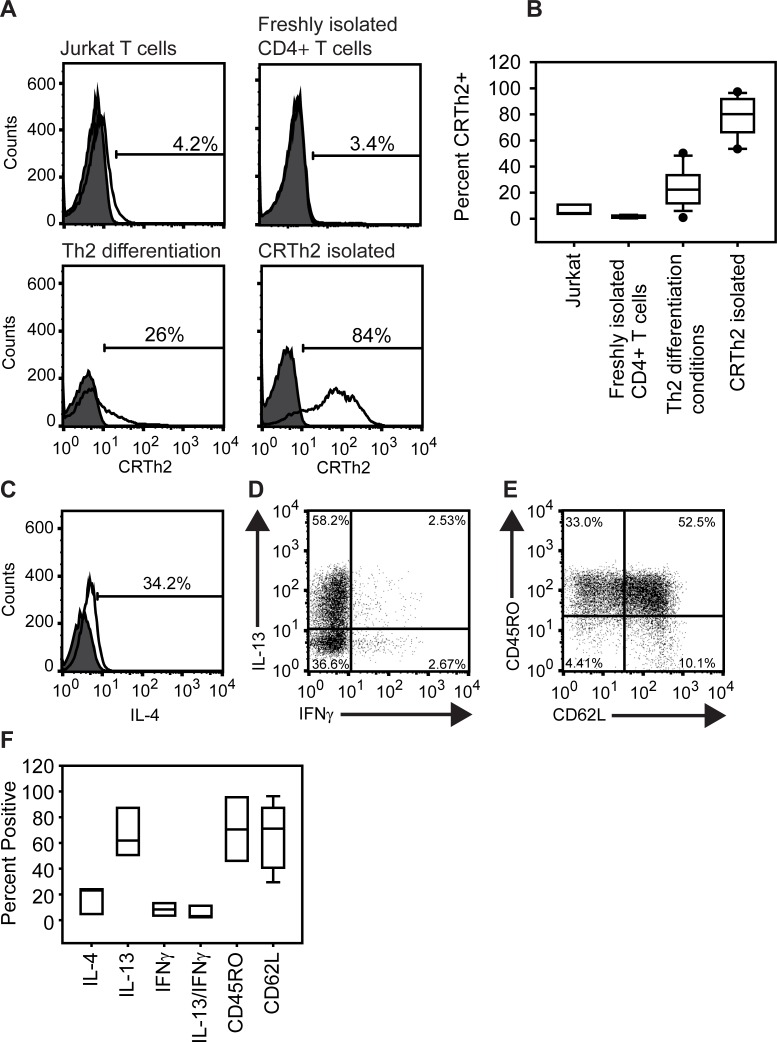
CRTh2 expression on Jurkat T cells and freshly isolated CD4^+^ cells. **(A)** Representative staining for CRTh2 staining in T cells. Empty histogram indicates CRTh2 staining and filled histogram indicates corresponding isotype control. Jurkat T cells and freshly isolated CD4^+^ cells express low levels of CRTh2 by surface staining. After 7 days in Th2 differentiation conditions, 24% of cells are CRTh2^+^. *In vitro* differentiated CRTh2^+^ Th2 cells express CRTh2 (78%) following the proliferation phase **(B)** Box and whisker plots summarizing CRTh2 surface staining from 3–18 experiments. (**C-E**) Using intracellular staining, CRTh2^+^ Th2 cells were IL-4 and IL-13 positive, but low IL-13 and IFNγ (2.5%) double positive **(D)** following mitogenic stimulation. *In vitro* differentiated CRTh2^+^ Th2 cells highly express CD45RO and CD62L **(E)** following the proliferation phase. **(F)** Box and whisker plots summarizing intracellular and surface staining from 3 independent cell lines.

Expression of CRTh2 is low on both the immortalized Jurkat T cell line (6.28% ± 3.92, n = 3) as well as human circulating CD4^+^ T cells (1.71% ± 0.97, n = 9; Fig 1A and 1B). Therefore, to study the expression and molecular regulation of *CRTh2* we generated CRTh2 expressing Th2 cell lines. Human CD4^+^ T cells were isolated from peripheral blood (>97% purity) and cultured in Th2 differentiating conditions for 7 days to induce expression of CRTh2 (23.79% ± 14.49, n = 18; Fig 1A and 1B). CRTh2^+^ cells were isolated providing enriched CRTh2-expressing cell lines (77.91% ± 14.73, n = 14; Fig 1A and 1B) exhibiting a polarized type 2 cytokine profile (Fig 1C, 1D and 1F). Isolated CRTh2^+^ cells were 17.33% positive for IL-4 (Fig 1C and 1F) and were predominantly IL13^hi^IFNγ^lo^ (Fig 1D and 1F) following mitogenic stimulation (4hrs). These cells also expressed CD45RO and CD62L (Fig 1E and 1F), indicating they developed into central memory T cells (T_CM_) like *in vivo* differentiated CD4^+^CRTh2^+^ T cells [8].

### CRTh2 expression is reduced after T cell receptor activation

**Fig 2:**
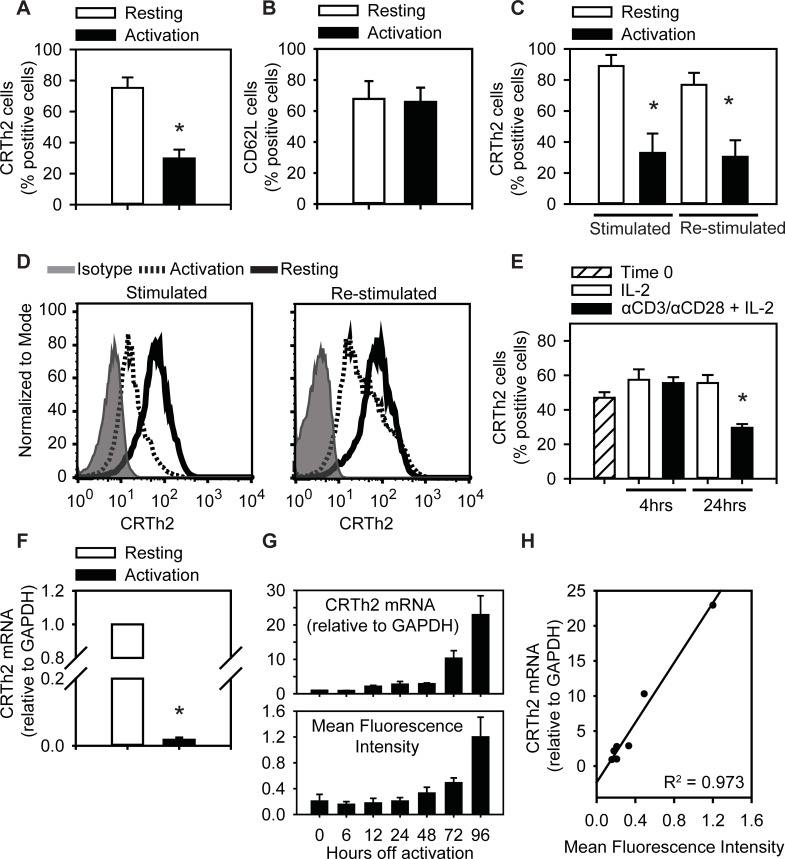
CRTh2 expression decreases following activation. **(A)** CRTh2 surface expression on *in vitro* differentiated CRTh2^+^ Th2 cells decreases following T cell stimulation (n = 8). (B) CD62L is equally expressed following the resting and activation phases **(**n = 6–8; 3 independent cell lines). (**C and D**) CRTh2 surface expression is dynamic, showing high (after resting) and low (after activation) over numerous cycles. **(E)** Surface CRTh2 expression was maintained after 4hr of stimulation but falls significantly by 24hr (n = 4). **(F)** CRTh2 mRNA is significantly decreased following 4hr of treatment with αCD3/αCD28. N = 3–4 independent experiments. (**G**) Following removal of TCR stimulation, mRNA (top) and surface staining (bottom—MFI to isotype surface expression by flow cytometry) for CRTh2 recovers over 3–4 days. (**H**) Return to surface expression of CRTh2 is highly correlated with mRNA levels. * p< 0.05, determined by Student’s t-test.

Since CRTh2 plays an active role in Th2 responses, we set out to study the regulation of this receptor. Th2 cell expression of CRTh2 was assessed after the resting (4 days culture in IL-2) and activation phase (3 days culture in IL-2 and anti-CD3 and anti-CD28) of culture. We found that the highest percentage of cells expressing CRTh2 was observed after IL-2 (76% +/- 6.8%), with a significant reduction after the activation phase (29% +/- 5.7%; Fig 2A). Interestingly, these same conditions did not alter the expression of CD62L which remained high following activation (Fig 2B). Our experiments also revealed that this regulation of CRTh2 was dynamic, as a pattern of high and low expression was consistently observed over multiple rounds of rest and activation (Fig 2C and 2D). We also performed a kinetic analysis, which showed that the percentage of CRTh2^+^ cells was reduced by 24 hours, but not 4 hours, after activation (Fig 2E). Transcription, however, exhibited a rapid response with the level of *CRTh2* mRNA levels significantly lowered after only 4 hours of activation (Fig 2F). Following removal of TCR activation with αCD3/αCD28, cells cultured in IL-2 alone gradually recovered CRTh2 expression at the level of mRNA as early as 12 hours (2-fold), while appreciable increase in surface protein took 48–72 hours (Fig 2G). Recovery of CRTh2 mRNA (relative to GAPDH) was strongly correlated with a concomitant increase in surface CRTh2 protein (Fig 2H)

### Characterizing transcription factor binding to the CRTh2 proximal promoter

**Fig 3:**
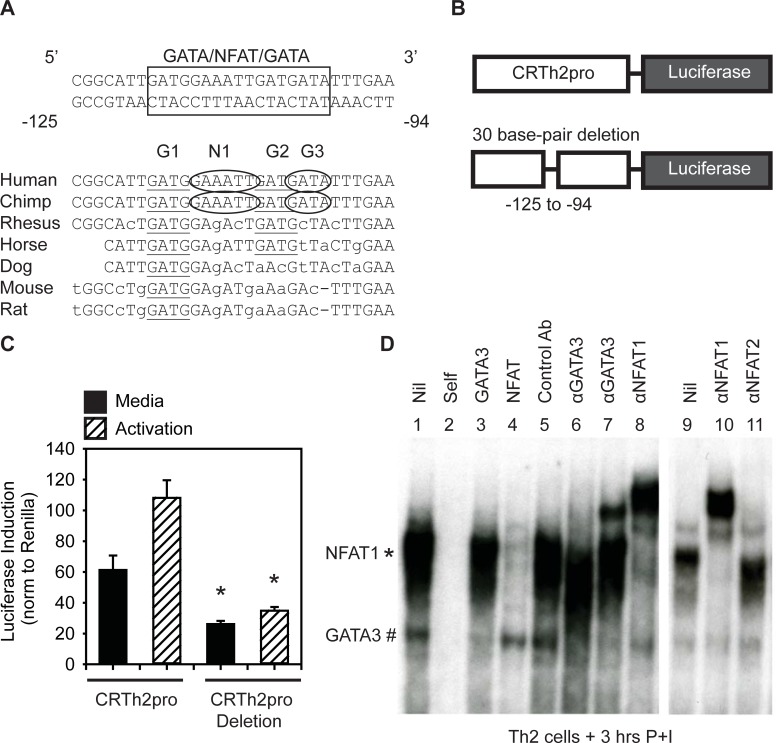
Predicted and observed transcription factor binding to the CRTh2 proximal promoter. **(A)** The *CRTh2* proximal promoter contains putative NFAT and GATA binding sites, which show varying degrees of evolutionary conservation. **(B)** Site-directed mutagenesis of the CRTh2pro reporter vector to delete the GATA/NFAT/GATA containing-region. **(C)** Specific deletion of the conserved (-125/-94) base pairs reduced both overall luciferase activity of the vector and response to activation with PMA/Ionomycin. **(D)** This region was used as a probe for EMSA. Nuclear extracts were made from *in vitro* differentiated CRTh2^+^ Th2 cells following the resting phase (IL-2) and were re-stimulated **(3h PI)**. NFAT1 and GATA3 bound to the *CRTh2* promoter, although NFAT1 binding is more prominent after PMA/ionomycin stimulation Competitors and super-shifting antibodies used are noted above the corresponding lanes. Antibodies denoted as α. Gels are representative of n = 8 from 6 independent cell lines. *p< 0.05, determined by Student’s t-test.

To examine the molecular mechanism for this downregulation, we used in silico analysis of the *PTGDR2* promoter to determine potential binding sites for transcription factors that may regulate CRTh2 expression. Inspection of the proximal promoter revealed a conserved region with overlapping GATA and NFAT binding sites (-125/-94, Fig 3A). We previously showed this region could bind recombinant GATA3 [43] and NFAT proteins have long been known to carry out TCR-mediated signaling [45, 46]. To further validate the function of this conserved region, we generated a luciferase reporter in which it was deleted (Fig 3B). We found this vector, that lacked both the GATA and NFAT binding sites (-125/- 94), had reduced baseline luciferase expression in media compared to the CRTh2pro/Luc as well as less activation in response to mitogenic stimulation (Fig 3C).

Therefore, we next assessed transcription factors binding at this site within the complexity of Th2 cell nuclear extracts using electromobility shift assays (EMSAs) with a probe representing this region of the *PTGDR2* promoter (Fig 3D). Fig 3D shows that we consistently observed 2 major complexes binding this probe (lane 1). Both were specific, as binding was competed with cold self-competitor (100X) (lane 2). The lower complex (#) was identified as GATA3, since it was competed with molar excess of a cold GATA3 consensus oligonucleotide (lane 3) and super-shifted (or missing) with two independent α-GATA3 antibodies (lane 6 and 7) but not an isotype matched control antibody (lane 5). The upper complex (*) was identified as NFAT1, since it was competed with molar excess of cold NFAT consensus oligonucleotide (lane 4) and super-shifted by an NFAT1 antibody (lanes 8 and 10), but not an NFAT2 antibody (lane 11). Together these data show that both GATA3 and NFAT1 bind this region of the *CRTh2* promoter following T cell activation.

**Fig 4:**
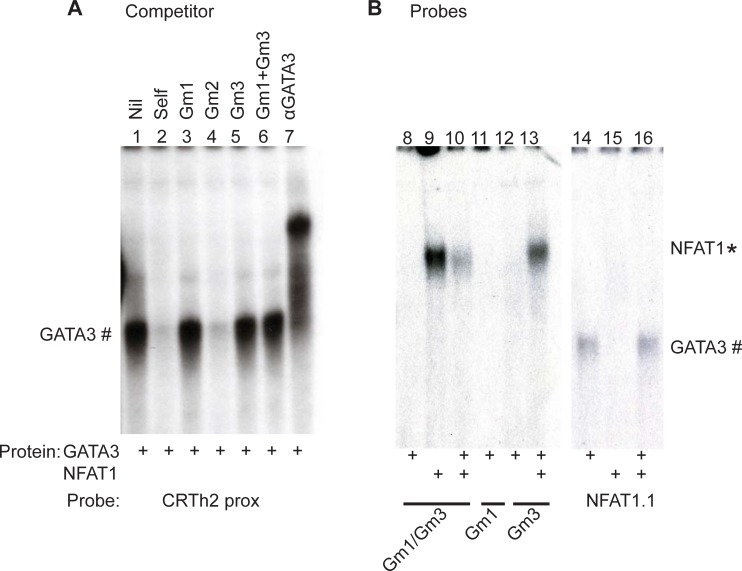
Determining the functional GATA3 sites in the CRTh2 proximal promoter. Recombinant GATA3 and NFAT1 were used to characterize binding. Mutated CRTh2 proximal oligonucleotides were used as competitors **(A)** and labelled as probes **(B)**. Competitors and super-shifting antibodies used are noted above the corresponding lanes. Antibodies denoted as α. G1, G2, and G3 refer to the GATA3 site that has been mutated in each case.

The architecture of this region of the *CRTh2* proximal promoter, consisting of an NFAT site flanked by 3 GATA sites (Fig 3A), indicated these factors may bind co-operatively (and enhance) or competitively (and inhibit) promoter activity. To determine the effect of both factors binding together, we generated *CRTh2* promoter oligonucleotides containing mutations in the predicted GATA3 sites (Table 1). We first used the oligonucleotides as cold competitors, pretreating the extracts with 100 times more (100X) than the probe, to determine their ability to compete binding. Recombinant human (rh) GATA3 binding was observed (Fig 4A; lane 1) and competed with cold self-competitor (100X, lane 2) and super-shift (lane 7). The importance of the G1, G2 and G3 sites were determined with rhGATA3 incubated with cold competitors (100X) of the mutated *CRTh2* proximal oligonucleotides (Table 1); Gm1 (G1 site mutation) (lane 3), Gm2 (G2 site mutation) (lane 4), Gm3 (G3 site mutation) (lane 5), or a double mutant Gm1 + m3 (G1 and G3 site mutations) (lane 6). Since Gm1, Gm3 and the two mutations combined (Gm1 + m3) were unable to compete, these sites appear necessary for GATA3 binding. Further, since mutation of one site did not compensate for the other non-mutated site (lanes 3, 5 and 6), both G1 and G3 sites appear necessary for GATA3 binding.

To determine the importance of the GATA sites for NFAT1 binding, the Gm1-Gm3 mutated CRTh2 oligonucleotides were labeled and used as probes. Fig 4B shows that rhGATA3 cannot bind to the Gm1/Gm3 mutant probe (lane 8), while rhNFAT1 can bind (lanes 9), indicating that NFAT1 binding does not require GATA3. GATA3 binding to Gm1 and Gm3 was also lost (lanes 11 and 12), though NFAT1 was still able to bind Gm3 (lane 13). We also observed that GATA3 could bind to the NFAT1 mutant (NFAT1.1, lanes 14 and 16), though NFAT1 could not (lane 15). When both GATA3 and NFAT1 were added, no low mobility band was observed, suggesting GATA3 and NFAT1 do not bind as a complex, although the Gm1/Gm3 probe appeared to bind NFAT1 with lower affinity (lane 10) than just the Gm3 probe (lane 13), suggesting that mutating both sites influenced NFAT1 binding. Collectively, these data show that GATA3 and NFAT1 bind independently to this region of the *CRTh2* promoter.

### NFAT1 reduces transcriptional activity of the CRTh2 proximal promoter

**Fig 5:**
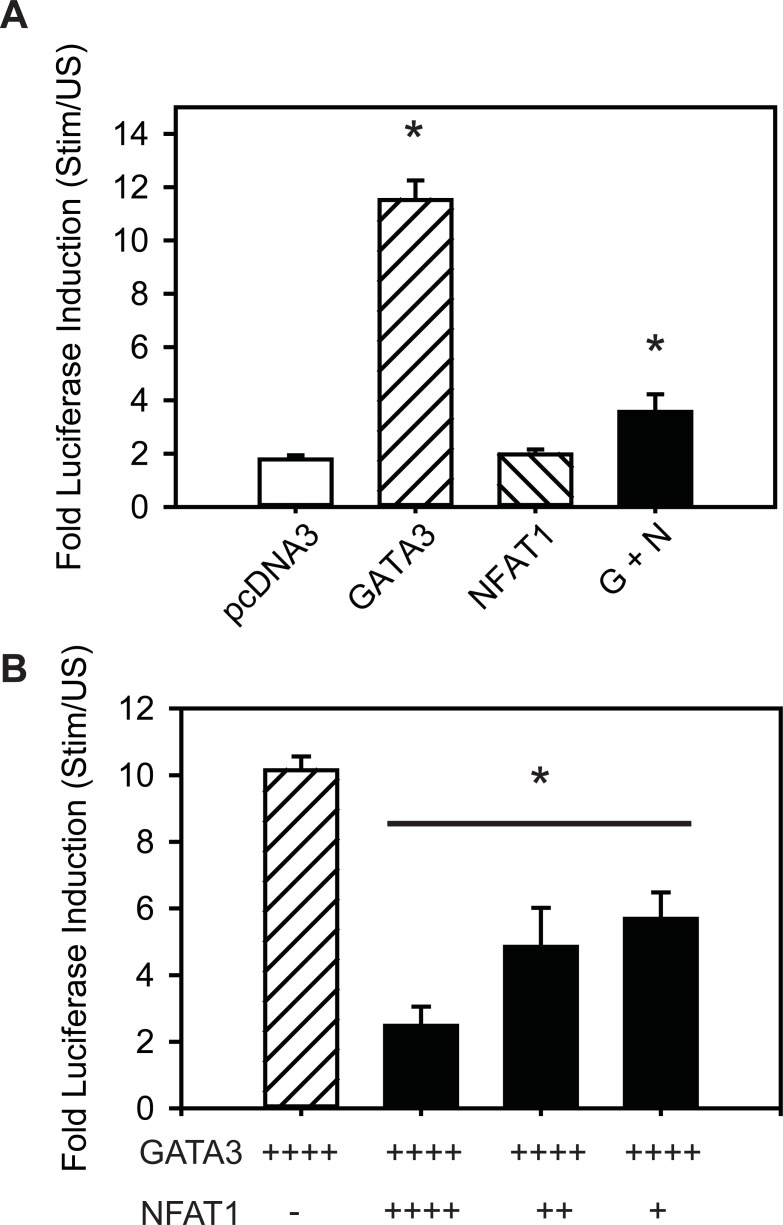
NFAT1 inhibits GATA3-induced *CRTh2* promoter activation. **(A)** GATA3, but not NFAT1, over-expression in Jurkat T cells increased transcriptional activity of CRTh2-pro/Luc. When GATA3 (G) and NFAT1 (N) were co-expressed this led to inhibition of GATA3-mediated activity. **(B)** Transcriptional activity of the *CRTh2* proximal promoter (CRTh2-pro/Luc) was measured by fold increase of relative luciferase activity (RLA) in stimulated (16h P/I) over unstimulated samples. When NFAT1 was co-transfected in increasingly lower amounts (up to 1/8^th^ GATA3) *CRTh2* promoter activity was recovered n = 3–5, *p< 0.05, determined by ANOVA with post hoc analysis using Holm-Sidak method.

Based on our transcription factor analysis showing that both GATA3 and NFAT1 bind following stimulation and appear to do so independently, we wanted to assess their influence on *CRTh2* promoter activity. To test this, GATA3 and NFAT1 were over-expressed at molar equivalents by co-transfection of GATA3 or NFAT1 expression vectors with a *CRTh2* promoter reporter construct (*CRTh2*pro/Luc). Fig 5A shows that GATA3 increased promoter activity 6.5-fold over the control DNA (pcDNA3.1^+^**; p<0.05)**, while over-expression of NFAT1 had no enhancing effect. When both were co-transfected, NFAT1 inhibited the GATA3-induced promoter activation. To test the negative effect of NFAT1 on *CRTh2* promoter activity, CRTh2pro/Luc was co-transfected with GATA3 and increasingly less NFAT1 (to 1/8^th^ of GATA3). This experiment revealed that reducing the amount of NFAT1 resulted in *recovery* of GATA3-induced activation of the *CRTh2* promoter, though only ~50% of GATA3 alone (Fig 5B). Collectively, these data suggest that GATA3 and NFAT1 have opposing effects on *CRTh2* promoter activity.

### NFAT mediates loss of endogenous CRTh2 expression following TCR activation

**Fig 6:**
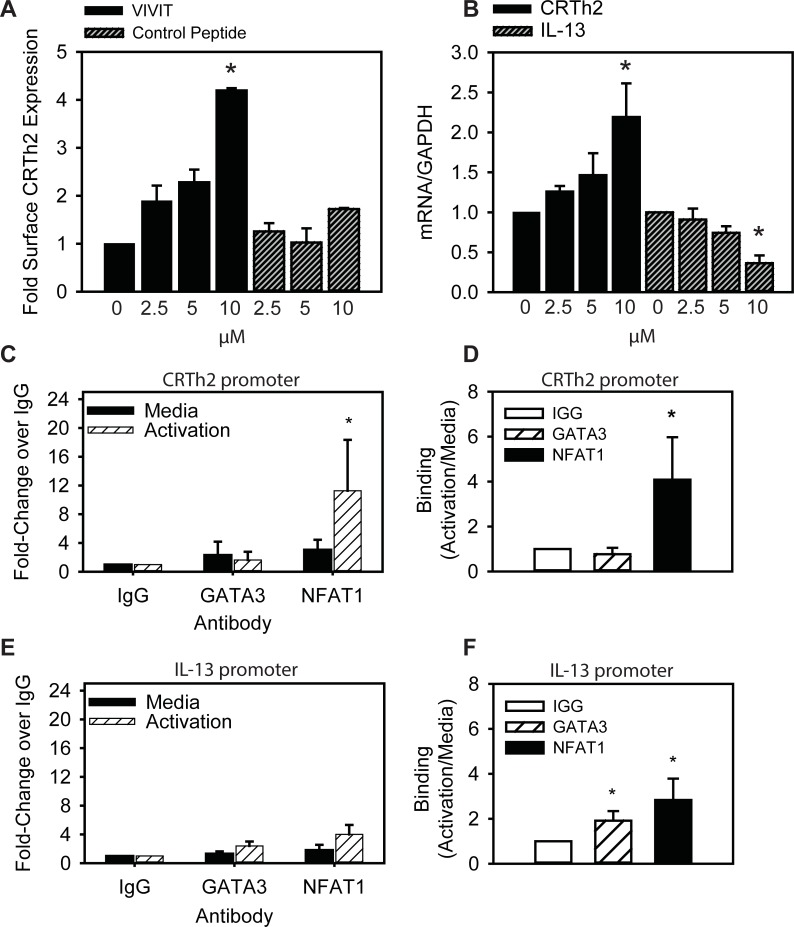
NFAT inhibition reverses the loss of CRTh2 expression following TCR stimulation. **(A)** Inhibiting NFAT with a cell permeable inhibitor peptide (VIVIT) maintains CRTh2 surface expression following 3 days of priming with αCD3/αCD28, control peptide (CP, VEET) did not affect the CRTh2 surface expression (n = 3). **(B)** CRTh2 mRNA was also recovered following 3 days of priming with αCD3/αCD28 in the presence of VIVIT, though IL-13 mRNA was increased. **(C-F)** GATA3 and NFAT1 binding to the promoters of CRTh2 and IL-13 was confirmed by ChIP (n = 4), *p< 0.05, determined by Student’s t-test, activation vs media.

Since T cell activation is associated with strong NFAT1 binding, yet reduced mRNA and surface expression of CRTh2, we next sought to inhibit nuclear translocation of NFAT to assess whether this would restore endogenous CRTh2 expression. To do this we used a cell permeable 30-mer peptide inhibitor of NFAT (11R-VIVIT), which binds and blocks the NFAT-calcineurin interaction, necessary for nuclear translocation. NFAT inhibitor or control peptide (11R-VEET) was added to Th2 cells during the 3 day activation phase (IL-2 and αCD3/CD28). Since we observed these conditions reduce CRTh2 expression (Fig 2A), this experiment was designed to detect whether blocking NFAT could interfere with the loss of surface CRTh2. Fig 6A shows that Th2 cells treated with the NFAT inhibitor had more CRTh2 surface expression (> 4-fold) compared to untreated cells and this effect was not observed in cells treated with control peptide. Similar results were observed for CRTh2 mRNA (> 2-fold; Fig 6B). In contrast to CRTh2, NFAT1 is known to induce Th2 cytokine expression [47, 48] and so we also examined the effect of VIVIT on IL-13 expression. Fig 6B also shows that increasing concentrations of the NFAT inhibitor resulted in decreased levels of IL-13 mRNA (> 50%). The differential effect of VIVIT on gene expression was substantiated by chromatin immunoprecipitation that show after activation only NFAT1 was bound to the CRTh2 promoter (Fig 6C and 6D), while both GATA3 and NFAT1 bound the IL-13 promoter (Fig 6E and 6F).

## Discussion

This study shows that expression of CRTh2 by CD4^+^ T cells is decreased following T cell activation through engagement of CD3 and CD28. While this observation was previously reported [4, 8], it received little attention. Our report is the first to investigate the underlying transcriptional regulation and to discuss the potential role of this mechanism in immune responses. Since both T cell activation and CRTh2 signaling induce Th2 cytokine expression [49], this was somewhat counterintuitive. However, our kinetic analysis revealed that while surface expression of CRTh2 was reduced by 24 hours, it was maintained up to 4 hours after activation, indicating there is a window of opportunity after activation in which CRTh2 remains on the surface of Th2 cells available to respond to PGD_2_.

The main source of PGD_2_ *in vivo* is considered to be mast cells following allergen/IgE crosslinking [2]. Release of PGD_2_ mediates chemotaxis of Th2 cells [1] orchestrating their infiltration to inflamed tissues [3, 50]. Our observation that TCR activation reduces CRTh2 expression is reminiscent of the ‘stop and go’ hypothesis that TCR and chemokine signaling compete for cellular control [51], with TCR downregulating chemokine receptors [52]. Indeed, PGD_2_ is also produced by dendritic cells and macrophages [3, 53] and so PGD_2_-CRTh2 mediated chemotaxis may draw and/or maintain Th2 cells within their proximity. Further, a study by Westhorpe *et*. *al*. [54] showed *in vivo* that activation of intravascular T cells by antigen-activated monocytes decreased migration and NFAT1 nuclear translocation. As such, our observation of TCR-mediated reduction of CRTh2 expression may reflect antigen-presenting cells (APC)-Th2 cell interactions that facilitate infiltration to the appropriate tissues. CRTh2 is a seven transmembrane G protein coupled receptor and PGD_2_ ligation signals through Gα_i_ [1] and both CRTh2 and TCR signaling trigger calcium release from intracellular stores [1, 55]. Sustained calcium signaling leads to anergy [55] and so downregulation of CRTh2 could be a way to mediate disengagement of Th2 cells from APCs, following a period of prolonged activation. Still yet, the CRTh2-PGD_2_ pathway activates PI3K and inhibits apoptosis [6] and so reducing CRTh2 may also mediate the resolution phase of inflammation.

Our characterization of *in vitro* differentiated Th2 cells showed they express both CD45RO and CD62L, indicating they are of central memory phenotype as they are *in vivo* [8]. While TCR signaling reduced CRTh2 levels, re-expression of mRNA was observed as early as 12 hours (2-fold), though the majority of the recovery of surface CRTh2 was between 72–96 hours. We believe this indicates expression of CRTh2 is dynamic and depends on extrinsic stimuli. CRTh2 has also been shown to mediate exit of T_CM_ cells from tissues to the lymph for drainage back to lymph nodes [35]. Therefore our observation that CRTh2 is re-expressed after recovery from antigen activation suggests that *in vivo* CRTh2 may also be important for Th2 cell trafficking and maintenance of a pool of memory Th2 cells.

CRTh2 expression is induced during Th2 cell differentiation [31]. This is presumably a direct result of GATA3 upregulation as Sundrud *et*. *al*. showed that over-expressing GATA3 induces surface expression of CRTh2 in naïve CD4^+^ T cells [42]. Within the innate lymphoid population, ILC2s are also defined by CRTh2 expression [13] and GATA3 has been shown to upregulate CRTh2 in these cells [15]. Other than this, little is known regarding the molecular regulation of CRTh2 expression or how GATA3 activates the *CRTh2* promoter. Our analysis revealed that deletion of a GATA/NFAT/GATA motif within the CRTh2 promoter significantly downregulated overall promoter activity, that over-expression of GATA3 increased *CRTh2* promoter activity and that two of the three putative sites (G1 and G3) were necessary for GATA3 binding. While this analysis suggests the G2 site may be dispensable, it is possible it increases affinity or influences binding in some way not observable by EMSA. Indeed, a study of the IL-13 promoter, which contains three tandem GATA3 sites (within 35 bp), showed that optimal binding and expression depended on all three sites being intact [56]. GATA3 can either dimerize to bind to adjacent sites (5 bp apart) or a single GATA3 molecule can bind to 2 sites within 3 bps [57]. In the *CRTh2* proximal promoter the GATA3 sites are 10 bp apart, suggesting that dimerization may be necessary. Though GATA3 is expressed by all human T cells, only high level GATA3 expression is associated with induction of CRTh2 [41]. This suggests that the number of GATA3 sites occupied, which would depend on the level of GATA3, may represent a regulatory mechanism for CRTh2 expression. Within CD4^+^ T cells, CRTh2 is specific to Th2 cells in humans [10], but not mice [58]. This could be due to the *CRTh2* promoter acquiring GATA3 sites over evolution, as only the G1 site was found in murine sequence, while the human promoter contains 2 more GATA sites (G2 and G3).

**Fig 7:**
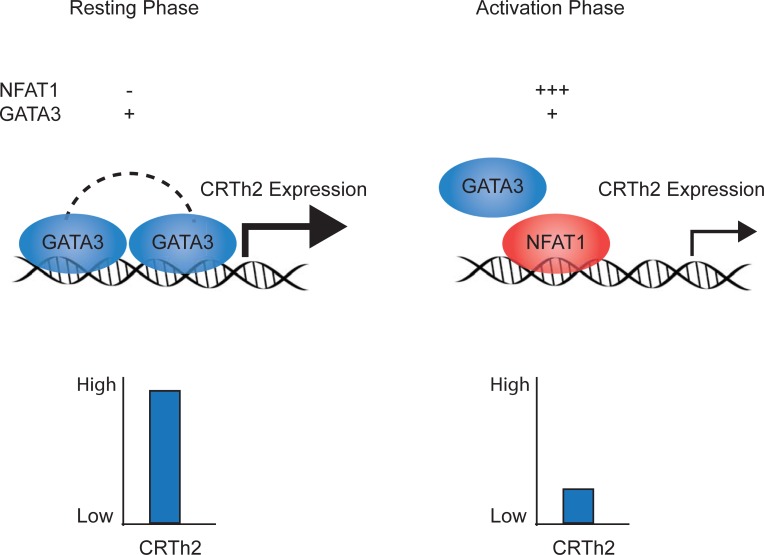
Schematic representation of molecular regulation of CRTh2. Although GATA3 can bind the *CRTh2* promoter probe, NFAT1 binds with greater intensity following activation and displaces GATA3, resulting in lower CRTh2 expression. Dashed line represents that one or two GATA3 molecules may bind.

Our study indicates that NFAT1 and GATA3 bind independently to this region of the *CRTh2* promoter. Co-expressing these factors showed that GATA3 induction of *CRTh2* promoter activity was inhibited by NFAT1. Reducing NFAT1 levels, either by transfecting fewer copies of expression vector or inhibiting activation and nuclear translocation with VIVIT [44, 59], was associated with recovery of both *CRTh2* promoter activity and endogenous CRTh2 expression. Collectively, these data suggest that the level of nuclear NFAT1 available to bind the CRTh2 promoter is increased as a result of T cell activation and interferes with GATA3-mediated expression of CRTh2 (Fig 7). Our findings are in line with the vast body of evidence suggesting NFAT1 exerts an overall negative regulatory influence on immune responses. Indeed, NFAT1^-/-^ mice showed hyper-proliferation, enhanced primary and secondary responses to Leishmania major *in vitro* and increased Th2 cell differentiation [60–62]. Heightened allergic inflammatory responses (eosinophils and IgE) [60], airway hyper-responsiveness in a model of allergic airways disease [63] and NFAT1 inhibition of the *CX3CR1* promoter following IL-15 stimulation have also been observed [64]. However, our results differ from a number of studies showing that GATA3 and NFAT1 binding together positively influences transcription in genes such as IL-13 [48, 65], CD40 [66] and ICOS [67]. As such, we believe our report is the first to show NFAT1 inhibiting GATA3-mediated activation. A caveat is that we used a peptide (VIVIT) that inhibits interaction of all NFAT family members with calcineurin [44, 59], and so we cannot rule out that recovery of CRTh2 expression may have involved other NFAT proteins, such as NFAT4 that has also been shown to suppress Th2 cytokine production [68].

In contrast to CRTh2 expression, we found that VIVIT reduced IL-13 mRNA levels, highlighting the complex role of NFAT1 in mediating Th2 cytokine expression. Indeed, many studies show NFAT1-/- mice exhibit enhanced type 2 cytokine expression [62, 69], due to compensation from other NFAT family members and effects on Th2 cell maturation [70, 71]. However, other studies show that NFAT1 induces IL-13 [72] in combination with GATA3 binding [48, 65]. Our data support this cooperative interaction, as we found both GATA3 and NFAT1 binding the IL-13 promoter and that inhibiting NFAT nuclear translocation with VIVIT reduced IL-13 mRNA levels.

Expressing NFAT1 at an equimolar concentration to GATA3 shut down *CRTh2* promoter activity. In fact, even when NFAT1 levels were reduced to 1/8^th^ of GATA3, the activity of CRTh2 was lower than when GATA3 was unopposed, suggesting NFAT1 serves to tone down the level of CRTh2 transcription. Looking at endogenous expression of CRTh2, the correlation between CRTh2 mRNA and protein during recovery of expression further supports the observation that CRTh2 expression appears to be under tight control. On the other hand, our kinetic analysis of downregulation of CRTh2 showed that transcript levels were significantly reduced (> 90%) by four hours. This rapid reduction could suggest CRTh2 is also subject to post-transcriptional regulation. Indeed, Huang et al. have shown that genetic variation within the 3’ untranslated region of CRTh2 influences transcript stability [73].

In conclusion, T cell activation reduces expression of CRTh2 at the level of both transcription and protein expression. This reduction correlated with NFAT1 binding to the *CRTh2* proximal promoter region, likely interfering with GATA3-induced activation (Fig 7). Like TCR crosslinking, CRTh2 activation increases intracellular calcium and so downregulation of CRTh2 may represent a regulatory mechanism to mitigate the effects of over-activation. Re-expression of CRTh2 following removal of TCR activation could influence Th2 cell emigration through lymphatic vessels and their transit back to the lymph nodes. These data illustrate the dynamic regulation of CRTh2, which may facilitate its role in the persistence of Th2 cells and allergic disease.
